# Creating the Map of Interactive Services Aiding and Assisting Persons With Disabilities (MSAADA) Project: Tutorial for the Novel Use of a Store Locator App

**DOI:** 10.2196/37036

**Published:** 2022-12-08

**Authors:** Mary Ann Etling, Michael Musili, Kaytlin Eastes, Eren Oyungu, Megan S McHenry

**Affiliations:** 1 Department of Pediatrics Indiana University School of Medicine Indianapolis, IN United States; 2 Richard M Fairbanks School of Public Health Indiana University Indianapolis, IN United States; 3 Moi University School of Medicine Eldoret Kenya; 4 Center for Global Health Indiana University Indianapolis, IN United States; 5 Department of Medical Physiology Moi University School of Medicine Eldoret Kenya; 6 Academic Model Providing Access to Healthcare Eldoret Kenya

**Keywords:** map, virtual, interactive, disability, resources, inclusion, mHealth, Kenya, global health, public health

## Abstract

**Background:**

An estimated 15% of the global population is living with a disability. In Kenya, children with disabilities remain among the most vulnerable populations, experiencing substantial barriers to wellness and inclusion. Smartphone ownership and internet access have been increasing across sub-Saharan Africa, including in Kenya. Despite these advances, online or mobile resources remain limited and difficult to find and navigate.

**Objective:**

This paper aims to describe the novel use of a store locator app to develop an interactive map of organizations that provide medical, educational, and socioeconomic resources to individuals with disabilities in Kenya. The target audience is individuals with disabilities, medical professionals, and organization leaders.

**Methods:**

A comprehensive list of organizations, government county offices, educational assessment and resource centers, and institutions was compiled. Organizations were contacted via email, WhatsApp, or in person for semistructured interviews. Based on the services offered, each organization was assigned categorical search tags. The data were entered into a third-party store locator app. The resulting map was inserted into a page on the Academic Model Providing Access to Healthcare (AMPATH) website.

**Results:**

The Map of Interactive Services Aiding and Assisting Persons With Disabilities (MSAADA; this abbreviation is also Swahili for “help”) was launched in July 2020 in both English and Swahili. The map included 89 organizations across Kenya. Of these, 51 were reached for an interview (for a 57% response rate). Interviewees cited limited paid staff and dependence on grant-based funding as primary challenges to growth and sustainability.

**Conclusions:**

MSAADA is an interactive, virtual map that aims to connect individuals with disabilities, medical professionals, and organization leaders to resources in Kenya. The novel use of a store locator app to compile resources in remote settings has the potential to improve access to health care for a wide variety of specialties and patient populations. Innovators in global health should consider the use of store locator apps to connect individuals to resources in regions with limited mapping.

## Introduction

### Background

According to the World Health Organization, an estimated 15% of the world’s population is living with a disability. Globally, individuals with disabilities have worse health outcomes, encounter more barriers to education and employment, and experience increased levels of poverty compared to individuals without disabilities [1]. Children with disabilities are among the most vulnerable populations. An estimated 11.2% of children and adolescents have a disability, and nearly 95% reside in low- and middle-income countries [2].

The prevalence of disability in Kenya reflects global trends. An estimated 10.3% of Kenyans have some form of disability, including difficulty with seeing, hearing, mobility, cognition, self-care, or communication [3]. In 2003, the government of Kenya passed the Persons with Disabilities Act, prohibiting discrimination against persons with disabilities and improving access to care, education, and employment [4]. Despite these legislative changes, children and adults with disabilities in Kenya continue to experience significant barriers to wellness and inclusion, including barriers to education [5], barriers to employment [6], and poorer health outcomes compared to individuals without disabilities [1].

Individuals with disabilities in sub-Saharan Africa experience multiple barriers to accessing health care and services. These barriers include stigmas associated with disability, a lack of awareness about disability, the inability to access transportation, and other poverty-related factors [7]. Few accessible resources exist to directly help individuals with disabilities navigate therapy and equipment needs. Additionally, due to limited availability, few health care professionals and skilled providers are knowledgeable about referral and equipment resources for families caring for individuals with disabilities [7,8]. Based on this understanding, we identified a need to compile resources in a centralized place where families could easily access them.

We recognized the growing share of Kenyans with access to smartphones and the internet as a potential pathway for connecting Kenyans to resources for individuals with disabilities. Smartphone ownership and internet access have been increasing across sub-Saharan Africa, including in Kenya [9]. Approximately 80% of Kenyans own a mobile phone, 30% own a smartphone, and 39% use the internet, including more than half of Kenyans between the ages of 18 and 29 years [9]. Despite these increases in access to information via the internet, many online resources are difficult to find and navigate.

### Objectives

This paper aims to describe the novel use of a store locator app to develop an interactive map of organizations that provide medical, educational, and socioeconomic resources to individuals with disabilities in Kenya. The project is titled MSAADA (Map of Interactive Services Aiding and Assisting Persons With Disabilities); the Swahili word *msaada* translates to the English word “help.” The target audience is individuals with disabilities, medical professionals, and organization leaders with access to needed services.

## Methods

### Setting: the Academic Model Providing Access to Healthcare Project in Western Kenya

In 1990, the Indiana University School of Medicine began a partnership with Moi Teaching and Referral Hospital, located in Eldoret, Kenya. In 2001, other universities joined to establish a long-term partnership known as the Academic Model Providing Access to Healthcare (AMPATH). The mission of AMPATH is to address needs for care, training, and research to improve the health of individuals globally. Within this tripartite mission, care remains most important and central to AMPATH. The model prioritizes the bilateral exchange of medical doctors, residents, students, and researchers to serve together in Kenya and North America. Every project based in Kenya must be care centered, sustainable, and locally led [10].

We are a team of 2 pediatricians (1 from Kenya, 1 from the United States) and 2 medical students (1 from Kenya, 1 from the United States). Much like other initiatives started within AMPATH, this project was formed to fill a gap in patient care. Health care providers at Moi Teaching and Referral Hospital were having difficulty connecting their patients with disabilities to available resources and services in their communities. The reasons for this were complex. In part, disability is an umbrella term for various conditions, meaning that a resource that supports one family may not be the best fit for another family. Additionally, Moi University Teaching and Referral Hospital is a large referral center, so providers in Eldoret may not be aware of resources for their patients in Kabsabet. In a setting where transport is limited, proximity is crucial. Finally, existing search engines and maps often do not contain updated or detailed information from organizations in Kenya; this missing information remains a major barrier to accessing these resources.

Ultimately, the team decided to make MSAADA available through the AMPATH website for two reasons. First, this academic partnership already has various long-term connections with organizations that could be added to the site. Many of the organizations that were added were known by the pediatric research team based in Kenya, given their experience living and working alongside children with disabilities and their families. Second, adding MSAADA to the AMPATH site allows for opportunities for trainees from both Kenyan and US institutions to help update the map over time. A critical component of this resource is that it is regularly updated to be sustainable. Having undergraduate and graduate students actively involved in this project allows for greater consistency overall.

### Creating a Database

The first step to developing MSAADA was to create a database of organizations, institutions, and government offices that provide resources for persons with disabilities in Kenya. This step was conducted using various methods. The team started with a list of organizations that were commonly known by the pediatric research team at Moi Teaching and Referral Hospital. Subsequently, the team used common search engines to start to find other organizations. With continued searching, the team was able to find several directories; however, many organizations’ contact information and services were not up-to-date. In some cases, the organizations in the directory no longer existed. The team compiled a comprehensive list. Organizations with an email address or WhatsApp number were contacted for a virtual interview to verify basic information and discuss the services provided by the organization. The information gathered included the organization’s name, address, email address, website URL, social media links, phone number, primary contact, longitude and latitude, services offered, payment options, requirements for enrollment, and transport services (Multimedia Appendix 1). After these interviews were conducted, a team member based in Kenya traveled to Nairobi to conduct interviews with other organizations in person. All information gathered in these interviews was stored in a shared database for the map.

### Selecting Storepoint: a Store Locator App

The next step was selecting a way to feature each of the resources in a single map that would be easy to use and manageable to update regularly. Storepoint is a user-friendly, customizable mapping feature that can be incorporated into any website. Its original purpose was as a store locator app that allowed businesses to display each of their locations on a map. It allows consumers to input their city name or zip code to find the nearest business location. It also has features on the map that allow consumers to filter locations based on specific characteristics. While Storepoint has a monthly cost, it is relatively intuitive to use and requires no prior knowledge or expertise in coding. After considering other comparable options, we chose Storepoint to host MSAADA, as it gave the team the greatest opportunity for customization without imposing the burden of having to train students in highly technical skills. From a sustainability standpoint, the cost to use Storepoint was allocated in the monthly research budget.

### Adding and Editing Organizations

The following steps detail how to add and edit organizations in Storepoint after gaining membership. On the main dashboard, users can select “Add Location” to add 1 location or “Bulk Import Locations” to use a spreadsheet to add multiple locations at once. For MSAADA, every location in Storepoint represents an organization. The fields on the form for organization name, email address, location, social media links (eg, Twitter, Facebook, and Instagram), photograph, logo, and “tags” (see the “Adding Search Categories” section of this paper) are filled in and these items are featured on the map. Location can be either determined by a street address or by coordinates for longitude and latitude; this choice provides flexibility and precision for organizations in this setting that do not have a traditional street address. To edit an organization, users can select “Search Locations” to find the organization they want to edit and click on it to update or change any information.

### Adding Search Categories

One useful feature of Storepoint is the ability to categorize organizations by various features that can help the user more easily locate a specific resource. This step involves “tagging” organizations within a “tag group.” Users can click a tag group drop-down menu in the search bar above the map and filter by a specific tag. For instance, there is a tag group called “Select by Cost” with the tags “$,” “Free,” “NHIF” (ie, the National Hospital Insurance Fund), and “Private Insurance.” Users can potentially click “Select by Cost” and then “Free” if they only want organizations that have free services to be shown on the map. Storepoint allows adding tags by selecting “Manage Locations” and then selecting “Tags and Filters.” To add a tag group, users click “Add A Tag Group” at the top. To add a tag to a location, users can add it to the information for a specific location under “Tags” (see the section “Adding and Editing Organizations” in this paper).

We created tag groups before we interviewed the organizations and adjusted our methods as the interviews progressed to reflect the types of resources offered by each organization. An unlimited number of tags and tag groups can be created and added for each organization. The tag groups and tags included for the initial phase of the map can be found in Multimedia Appendix 2. The filter can be set up with AND or OR operators when multiple tags are chosen. We have it set to AND for MSAADA. For example, a user might select “free” AND “speech and language therapy” AND “cerebral palsy” as tags. In this search, only organizations with all 3 of these tags would appear, meaning they offer free speech and language therapy for individuals with cerebral palsy. Since the map already has hundreds of organizations featured, this allows individuals to easily search for specific resources of interest.

### Embedding in the Website

This final step discusses how to incorporate a Storepoint map into a website. This step involves going to “Embed Locator” on the Storepoint dashboard. An embed link is provided that can be copied into the website HTML. Any changes made in the Storepoint account will automatically be reflected, so the embedded code does not need to be adjusted. For our team, this was an added benefit for this project, since we were not personally managing the website. AMPATH generously allowed the team to use a URL connected to their domain for MSAADA and helped us to create a functional site for users.

### Additional Features

**Figure 1:**
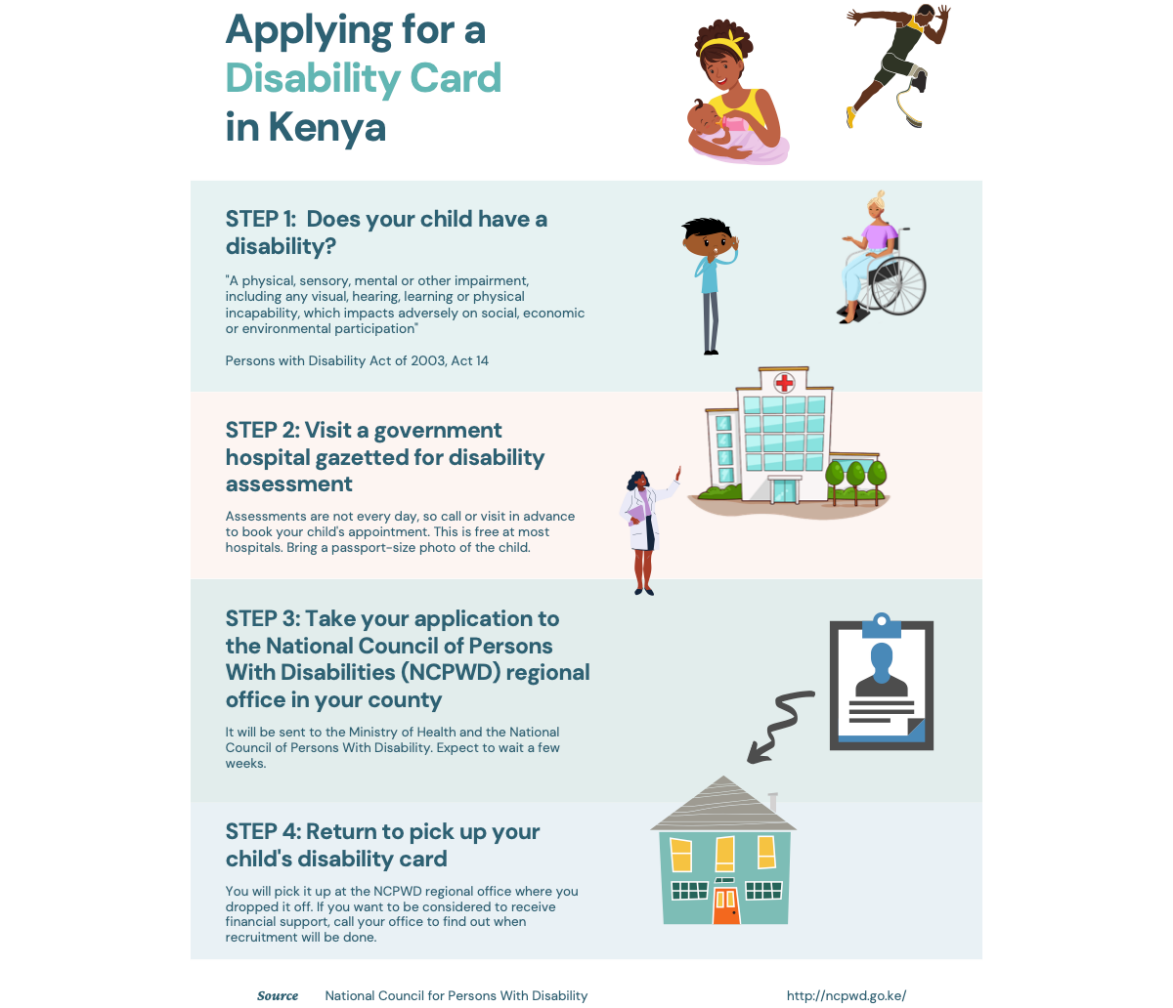
Representation of a downloadable step-by-step guide to obtaining a disability card in Kenya created for this study.

In addition to the interactive map, the web page provides access to 3 other valuable resources: a disability card infographic, Kenya Disability Resource, and Kenya Disability Directory. The disability card infographic was created by the team to explain and illustrate the process for obtaining the government card issued to individuals with disabilities in Kenya, as shown in Figure 1. This infographic was also translated to Swahili and made accessible on the website.

Kenya Disability Resource is a website dedicated to providing information, creating awareness, and building community around disability in Kenya. Kenya Disability Directory is a directory in the PDF format created by Handicap International in 2010 that includes hundreds of organizations.

### Functionality of MSAADA

Throughout the Methods section of this paper, the primary format has been a step-by-step tutorial on how to use the store locator app to develop an interactive resource map in a remote setting. Here, we will briefly describe how the target audience could use MSAADA. Any individual with internet access can log on to the map using the URL created by AMPATH to host the web page [11]. At the top of the page, users can select “Unazungumza Kiswahili?” which will take them to the same resource map translated into Swahili. If English is preferred, users can continue to the map.

**Figure 2:**
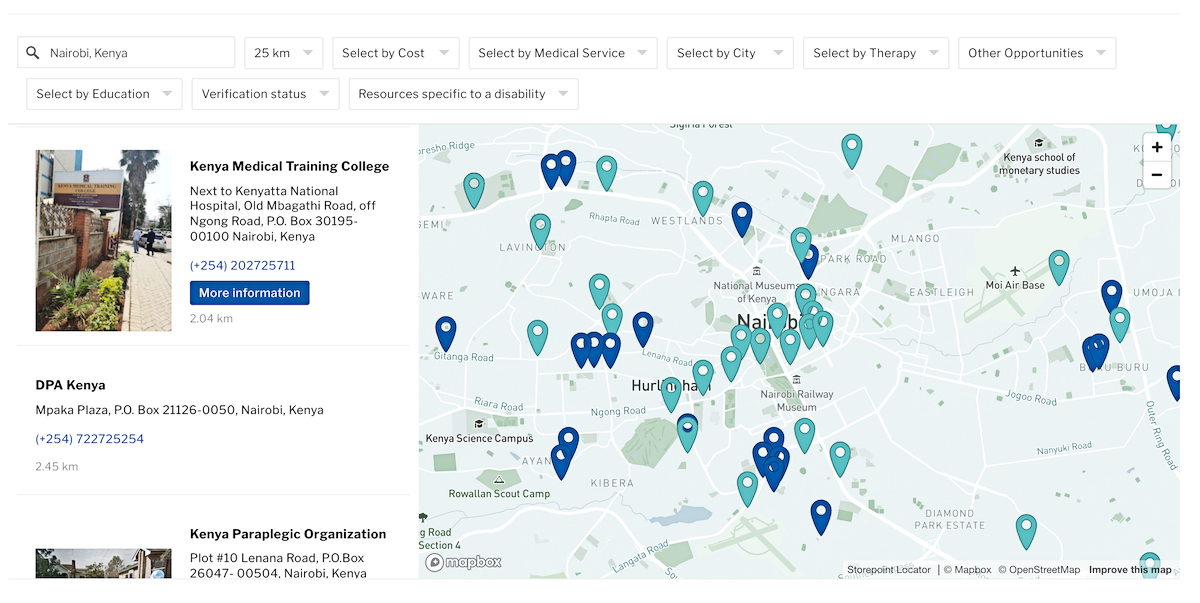
Screenshot of the interactive map with settings to show all resources within 25 km of Nairobi, as selected in the search bar at the top of the map. Map data from OpenStreetMap.

After scrolling down, users can type in their location by inputting their village, city, or region. The location search bar will auto-populate options to provide additional choices. They can then use the drop-down menu to choose how many kilometers they are willing to travel from their selected location, with options ranging from 5 to 100 kilometers. In Figure 2, “Nairobi, Kenya” has been typed in and “25 km” has been selected from the drop-down menu.

**Figure 3:**
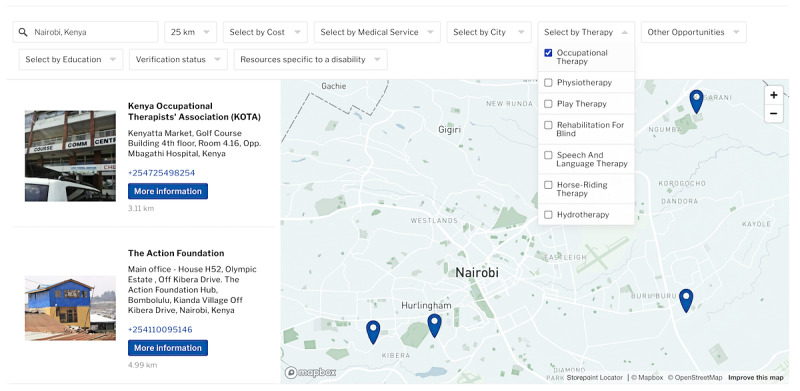
Screenshot of the interactive map with settings to show all resources that are verified and offer occupational therapy services within 25 kilometers of Nairobi. Map data from OpenStreetMap.

The remaining drop-down menus allow users to select from the various criteria listed earlier as tags. In this case, “verified,” under “verification status,” and “occupational therapy,” under “select by therapy,” have been selected, which will show all organizations that provide verified occupational therapy services within 25 kilometers of Nairobi. In this search, 4 organizations are shown, as seen in Figure 3.

## Results

MSAADA was launched in July 2020 in both English and Swahili. The team compiled a list of 219 organizations and institutions within Kenya that offered services to individuals with disabilities. Of these organizations, 89 had an email address or WhatsApp account to which the team was able to send an introductory email to set up an interview. Of the 89 organizations that were contacted, 12 agreed to conduct interviews. An additional 39 were reached in person and completed an interview. In total, interviews were conducted with 51 of the 89 organizations, giving a response rate of 57%. In the interviews, the organization representatives mentioned limited staff and volunteers and grant-based (ie, short-term) funding as the primary challenges to the growth and sustainability of their services. When asked about features that would be valuable for the site, the majority of organization representatives mentioned the need for it to be accessible to all individuals with disabilities.

## Discussion

### Advantages

#### Translation to Multiple Languages

One of the most valuable features of Storepoint is that the map can be translated into multiple languages. Each translation of the map has a unique embedded code that can be inserted into a website. The 2 official languages in Kenya are English and Swahili, but over 30 distinct languages and dialect clusters are also spoken. Originally, MSAADA was developed in English, and members of the team in Kenya then translated the site features and additional documents on the site into Swahili. Additionally, since the team originally created MSAADA, the AMPATH website has added an accessibility feature called UserWay, which is visible as a small widget icon in the lower right-hand portion of the screen. Among many accessibility features that are detailed later in this tutorial, this widget can translate the entire site into 42 additional languages.

#### New Organization and Feedback Forms

Another key feature of the website is a form for organizations not yet featured on the map to add their organization and provide key information. The site also includes a link to a form for organizations to provide feedback on the map and make updates to information specific to their organization. Upon submission, both surveys are automatically sent directly to an individual on the team who consistently updates the map. This feature allows new organizations and already featured organizations to ensure that the information on MSAADA is accurate and up-to-date.

### Disadvantages and Limitations

#### Grant-Based Funding

A major challenge noted by several organizations was that projects and services are highly dependent on grant funding. As a result, a specific service may only be offered for 1 to 5 years, based on the length of the grant. This presents an obstacle for both health care providers and individuals with disabilities because the resources that exist are constantly evolving. The benefit to having an interactive map is that changes can be quickly made by the team, and these changes are reflected immediately in MSAADA for its users.

#### Communication With Organizations

Another challenge for the development of MSAADA was communication with organizations, as seen with the low initial response rate. This is likely due to several factors, including outdated contact information, limited access to the internet, and limited time of staff or volunteers. During the interviews, many organizations noted they had a limited number of employees or were completely operated by volunteers, making it difficult for them to respond or devote time to an interview. This presented a challenge to obtaining information that was verified and accurate. Additionally, most of the communication was initially conducted via email, WhatsApp, and video chat from the United States. To further develop relationships with organizations, a member of the team based in Kenya visited 39 organizations in Nairobi to meet with employees and volunteers in person. This aided greatly in verifying contact information from several organizations within a short timeframe, but it may not be feasible for every organization across Kenya on an annual basis. However, we found that through this relationship-based model, we were able to gather updated information that could not be found online elsewhere.

#### Reliance on Human Endeavor

A final limitation of this model that should be noted is that it relies heavily on human endeavor to keep updated. To develop MSAADA, 1 research team member input the data collected in the virtual and in-person interviews and from the 2 Google forms. Updating will be done on a weekly basis by checking the 2 Google forms and making edits to the Storepoint page. In the future, this may be automated, but at this time, the human component allows for two major advantages: (1) we can build a relationship with the organization, and (2) we can review and revise any errors before uploading information to the public. Additionally, we have a sustainable internal system for updating the site. There are always at least 2 to 4 medical students or graduate students on the research team at any given time. Every semester, a student will be assigned the responsibility of working with Kenyan colleagues and organizations to maintain MSAADA.

### Future Improvements

#### Increased Accessibility and Inclusivity

A crucial component of MSAADA is that is remains an accessible and inclusive resource for all users. Currently, the AMPATH website uses UserWay, which includes a dictionary; screen reader; functions for contrast, highlighting, text sizing, and spacing; dyslexia-friendly options; and cursor formatting. These features improve usability for individuals who have visual impairments or difficulty reading written text on a computer screen. Another key accessibility feature is ensuring that the site remains financially accessible to users across Kenya. Thus, this resource will remain free for users to avoid excluding anyone based on socioeconomic status. Additionally, since the internet remains a major expense, a long-term goal is to investigate the possibility of creating a smartphone app version of MSAADA that could be downloaded and used without internet access. Finally, at this time, the map is limited to organizations and institutions within Kenya. To make it more geographically accessible, the map could be expanded to include locations in neighboring countries in East Africa, such as Uganda and Tanzania.

#### Information Guides

In the next phase of MSAADA, another goal is to have more detailed information about each resource that is organized for individuals with disabilities, medical professionals, and organization leaders. One proposal is to create clear, easy-to-refer-to information guides about each organization. For instance, a medical professional could use this guide to find free speech and language therapy for individuals with cerebral palsy and be able to provide the contact information, website, and address of the organization. Moreover, if the medical professional could use the information guide to direct the patient to a 1-page chart with more detailed information about when or how to qualify for the program, the guide could be a valuable extension of the map. These could be created in a standard format, translated to multiple languages, and uploaded as downloadable files to the web page.

#### Promotion and Evaluation

Another next step for MSAADA will be to seek feedback in order to evaluate the usability of the interactive map. The team plans to introduce MSAADA to groups of individuals in the target audience. A survey or short interview guide will be developed in order to gain feedback from individuals and to evaluate areas for improvement. Eventually, the team plans to use the “Locator Analytics” feature of Storepoint, which allows users to create a heat map of popular search areas, develop a chart of locator views and searches over time, and obtain the average distance individuals travel to their destination. By publishing this data, MSAADA will not only benefit those currently using it, but also provide organizations with data on how to better reach individuals with disabilities in the future.

### Conclusion

This paper describes the novel use of a store locator app to develop an interactive map of organizations that provide medical, educational, and socioeconomic resources to individuals with disabilities in Kenya. The use of a store locator app to compile resources in remote settings has the potential to improve access to health care for a wide variety of specialties and patient populations. Innovators in global health should consider the use of store locator apps to connect individuals to resources in regions with limited mapping.

## Appendix

Multimedia Appendix 1 Interview guide to verify organizations.

Multimedia Appendix 2 Search tags for organizations.

## Abbreviations

AMPATH: Academic Model Providing Access to Healthcare
MSAADA: Map of Interactive Services Aiding and Assisting Persons With Disabilities
