# Supplementary material for: Creating the Map of Interactive Services Aiding and Assisting Persons With Disabilities (MSAADA) Project: Tutorial for the Novel Use of a Store Locator App
Source: Interact J Med Res. 2022 Dec 8;11(2):e37036. doi: 10.2196/37036 (PMC9782326; doi:10.2196/37036)
Supplement: Multimedia Appendix 1 [file ijmr_v11i2e37036_app1.docx]

**Multimedia Appendix 1.** Interview Guide to Verify Organizations

Date of Interview:

Organization Name:

Interviewee:

Position:

### Could you tell us a little bit about your organization?

### What types of services or resources does it provide?

### What is your region of service?

How many employees and/or volunteers work at your organization?

What are the days and hours of operation? Are you open throughout the year?

What is the cost for your services? Do you subsidize or accept insurance?

Do you offer transportation?

Do you have a faith affiliation?

Are there any requirements for individuals to enroll in services?

Would you like to be featured on the MSAADA Interactive Resource Map?

Do you know of any other organizations providing resources or services to persons with disabilities?
