# Supplementary material for: Creating the Map of Interactive Services Aiding and Assisting Persons With Disabilities (MSAADA) Project: Tutorial for the Novel Use of a Store Locator App
Source: Interact J Med Res. 2022 Dec 8;11(2):e37036. doi: 10.2196/37036 (PMC9782326; doi:10.2196/37036)
Supplement: Multimedia Appendix 2 [file ijmr_v11i2e37036_app2.docx]

**Multimedia Appendix 2.** Search Tags for Organizations

“Tag group” 🡪 “Tag A”, “Tag B”, “Tag C”, “Tag D”

Select by Cost 🡪 $, Private Insurance, NHIF, Free

Select by Medical Service 🡪 Medical Referral, Mobility Device, Surgery, Medical Care, Dental Care, Ear Impressions

Select by City 🡪 Mombasa, Nairobi, Nakuru, Machakos, Kisumu, Busia, Kisii, Eldoret, Embu

Select by Therapy 🡪 Occupational Therapy, Physiotherapy, Play Therapy, Rehabilitation for Blind, Speech and Language Therapy, Horse-Riding Therapy, Hydrotherapy

Select by Education 🡪 Sibling Training, Special Education Training, Braille Center, Caregiver Training, Disability-Inclusive School, Vocational Training

Select by Disability 🡪 Vision-Impaired, Deaf, Albino, Autism, Cerebral Palsy, Club Foot

Other Opportunities 🡪 Research, Toys, Advocacy, Childcare, Dance, Female Mentorship, Income-Generating Activity

Verification Status 🡪 Unverified, Verified
